# Current Insights into 3D Bioprinting: An Advanced Approach for Eye Tissue Regeneration

**DOI:** 10.3390/pharmaceutics13030308

**Published:** 2021-02-26

**Authors:** Sandra Ruiz-Alonso, Ilia Villate-Beitia, Idoia Gallego, Markel Lafuente-Merchan, Gustavo Puras, Laura Saenz-del-Burgo, José Luis Pedraz

**Affiliations:** 1 NanoBioCel Research Group, Laboratory of Pharmacy and Pharmaceutical Technology, Department of Pharmacy and Food Science, Faculty of Pharmacy, University of the Basque Country (UPV/EHU), Paseo de la Universidad 7, 01006 Vitoria-Gasteiz, Spain; sandra.ruiz@ehu.eus (S.R.-A.); aneilia.villate@ehu.eus (I.V.-B.); idoia.gallego@ehu.eus (I.G.); mlafuente004@ikasle.ehu.eus (M.L.-M.); gustavo.puras@ehu.eus (G.P.); 2 Networking Research Centre of Bioengineering, Biomaterials and Nanomedicine (CIBER-BBN), Institute of Health Carlos III, 28029 Madrid, Spain; 3 Bioaraba, NanoBioCel Research Group, 01009 Vitoria-Gasteiz, Spain

**Keywords:** 3D bioprinting, cornea, retina, ophthalmology, tissue regeneration

## Abstract

Three-dimensional (3D) printing is a game changer technology that holds great promise for a wide variety of biomedical applications, including ophthalmology. Through this emerging technique, specific eye tissues can be custom-fabricated in a flexible and automated way, incorporating different cell types and biomaterials in precise anatomical 3D geometries. However, and despite the great progress and possibilities generated in recent years, there are still challenges to overcome that jeopardize its clinical application in regular practice. The main goal of this review is to provide an in-depth understanding of the current status and implementation of 3D bioprinting technology in the ophthalmology field in order to manufacture relevant tissues such as cornea, retina and conjunctiva. Special attention is paid to the description of the most commonly employed bioprinting methods, and the most relevant eye tissue engineering studies performed by 3D bioprinting technology at preclinical level. In addition, other relevant issues related to use of 3D bioprinting for ocular drug delivery, as well as both ethical and regulatory aspects, are analyzed. Through this review, we aim to raise awareness among the research community and report recent advances and future directions in order to apply this advanced therapy in the eye tissue regeneration field.

## 1. Introduction

**Figure 1:**
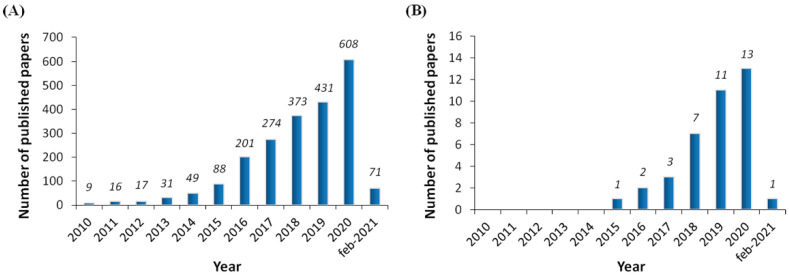
Histogram showing the timeline of publications (number per year), from the PubMed website, using the key “3D bioprinting” (**A**), and “eye/ocular/cornea/retina 3D bioprinting” (**B**) (updated to 2 February 2021).

The development of new revolutionary technologies during recent years, such as the use of Big Data, virtual reality systems and three-dimensional (3D) bioprinting, has created great expectations in the scientific community, not only regarding the improvement of the quality of life in patients affected by devastating pathologies, but also in terms of saving health-care associated resources [1,2,3]. In this regard, 3D bioprinting is an emerging manufacturing technology which holds great promise for a wide variety of biomedical applications, including drug testing, pathophysiological studies and regenerative medicine [4]. Specific benefits of such a technology would include the development of more targeted and personalized therapeutic approaches, as well as the possibility of obtaining functional models of tissues and organs for research purposes, increased reproducibility and higher capacity for drug lab testing studies, etc. In addition, for regenerative medicine applications, key advantages include automated tissue fabrication and the flexibility of incorporating many different materials and cell types in precise anatomical 3D geometries [4]. Consequently, the interest and investment in this promising technology has dramatically increased during the last five years [5] and, in fact, there is a clear exponential tendency regarding the number of published papers in the last decade (Figure 1A).

From a conceptual standpoint, 3D bioprinting refers to the “additive manufacturing” process based on a layer-by-layer approach with the deposition of bio-inks in a precise spatial arrangement [6], which makes it a suitable technology for obtaining highly complex structures similar to the original tissues of the eye [7]. The bio-ink is composed of structural components or biomaterials, crosslinkers, functional elements and living cells. Compared to non-biological printing, which has been proved suitable for the manufacturing of medical devices and patient-tailored prosthetics for more than 30 years [8], 3D bioprinting involves additional challenges and requires the multidisciplinary integration of different technological and medical fields. These complexities refer mainly to the selection of biocompatible materials, cell types, biomechanical cues and the overcoming of technical difficulties due to the sensitivities of living cells [9]. By using different materials and designs, the structural, physicochemical and mechanical properties of the bio-printed structure can be adjusted [10]. In general terms, the fundamental aspects that need to be taken into account for an appropriate selection of biomaterials include their printability, biocompatibility, degradation kinetics and byproducts, structural and mechanical properties, and biomimicry. Nowadays, for eye tissue regeneration applications, the most commonly used biomaterials in bio-inks are based on naturally derived polymers such as collagen, gelatin, alginate or hyaluronic acid [11], whose similarity to human extracellular matrix (ECM) and inherent bioactivity represent a relevant advantage [9]. Additional to the selection of suitable biomaterials, the target tissue determines the type of cells that need to be used in the bio-ink. In most cases, the tissues of the eye are made up of more than one cell type. For this reason, many of the approaches made to date incorporate more than one cell type or contemplate, as future steps, the incorporation of more cell types in the obtained scaffolds.

**Figure 2:**
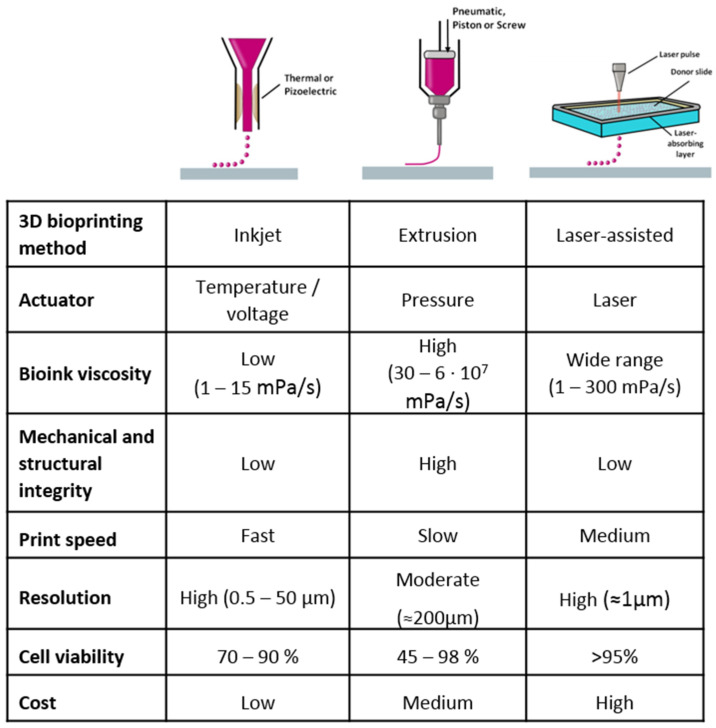
Major three-dimensional bioprinting techniques and their specific features.

In 3D bioprinting, the three major techniques include inkjet bioprinting, based on the deposition of bio-ink droplets, laser-assisted bioprinting, based on laser stimulation, and extrusion bioprinting, which uses mechanical force to deposit a continuous flow of bio-ink, each presenting specific features [4]. The choice of the most suitable approach depends on the specific application of interest, since each 3D printing technique holds its own peculiarities and these result in different outcomes [12,13,14,15,16,17] (Figure 2).

Some success has been demonstrated in early attempts to recreate complex tissue structures, and latest research evidences significant improvement in terms of effectiveness, resolution, accuracy and manufacturing speed of this customizable technique. All of this suggests, from a technical point of view, that the fabrication of biocompatible and functionalized full bio-printed organs will be possible in the near future [18]. In this regard, the success of 3D bioprinting for tissues and organs also depends on the target organ or tissue. For instance, for structurally simple tissues such as skin, 3D bioprinting is close to becoming a clinically relevant method for producing skin grafts and is already being used in the cosmetic industry [4]. On the other hand, for more complex organs such as the heart, it becomes more challenging to precisely reproduce its diverse functionality and heterogeneous composition, so is still far from clinical translation [4].

The field of ophthalmological applications of 3D bioprinting is also gaining interest due to the specific features of the eye (Figure 1B). In addition, its anatomical disposition provides easy access for surgery and implantation in both the inner layer and overall in the anterior chamber. Furthermore, the privileged immune condition of the eye coupled with the disposition of multiple diagnostic tools, turn it into an attractive organ for the application of 3D bioprinting technology, reducing the lack of organ donors along with the problems associated with rejection of transplanted grafts due to inappropriate immune response [19]. Some of the ophthalmological applications are the development of 3D bioprinting of anatomically realistic ocular models to enhance both education and clinical practice, providing better training opportunities, or the design of cost-effective personalized approaches by manufacturing specific ocular structures to treat serious eye diseases that affect more than 30% of the world’s population [20]. Depending on the specific eye disease to be treated, different ocular structures can be 3D bio-printed taking into account each disease’s specific structural and functional complexities.

As known, the eye is a complex, isolated, highly evolved organ with different and specifically organized tissue structures, which embrace, from an anatomical point of view, “simple” cell multilayer structures such as the cornea and more complex structures enclosing central nervous system cells, such as the retina. However, it is noteworthy that each eye tissue possesses its complexities when implementing 3D bioprinting. In this regard, the cornea represents a key ocular transparent tissue for vision that can be bio-printed, due its relatively simple structural disposition, into five independent layers. In normal conditions, the different hydrophilic and hydrophobic permeability of such layers, along with the interconnected tight junctions in epithelial cells, can severely limit the access of external agents into inner ocular structures, acting as a potent biological barrier [21]. However, severe alterations in the corneal thickness along with keratitis of multiple causes can lead to advanced stages of corneal tissue damage, where corneal transplant is the last medical choice. In this scenario, 3D bio-printed corneal tissue could emerge as an interesting medical option, bearing in mind that severe corneal disorders are the main cause of blindness worldwide [20]. In addition, it is worth mentioning that nowadays there is a lack of full-structure corneal in vitro models for both drug screening and toxicological testing, and that the deficit of donated corneas has increased the urgency of transplantable corneal substitutes. On the other hand, there are other relevant ocular tissues such as the retina, where the application of 3D bioprinting technology is more challenging from a scientific point of view, due to its complexity, neurological origin and connections with other areas of the central nervous system [22]. Some retinal dystrophies that dramatically hamper human life and for which there are no currently effective treatments include retinitis pigmentosa, Stargardt disease and age-related macular degeneration, to name just the most representative. Therefore, the possibility of bio-printing functional retinal tissue for both drug testing and graft implantation purposes has recently caught the attention of the scientific community in order to offer an alternative medical approach against such devastating retinal pathologies.

In any case, the field of 3D bioprinting for regenerative medicine is in its early stages and there are still several technological challenges to overcome, as well as some relevant ethical and regulatory concerns to be addressed, before fabricating large scale organs of all levels of complexity [23]. For instance, to become a realistic medical option, several parameters still need to be deeply considered such as the biocompatibility and mechanical properties of engrafts along with the biological behavior and attachment properties of cells, to name just the most relevant [22]. Nevertheless, if improvement and evolution in 3D bioprinting technology continues to progress exponentially, the creation of artificial biosynthetic and customizable full eyeballs in the near future is likely, along with other applications that have not yet been fathomed [24,25].

This review further discusses the current status and implementation of 3D bioprinting technology in the ophthalmology field in order to manufacture relevant tissue-engineering items such as the cornea, retina and conjunctiva. Special attention will be paid to the description of the most commonly employed bioprinting methods, and the most relevant eye tissue engineering studies performed by 3D bioprinting technology at preclinical level. In addition, regulatory, ethical and future directions related to the use of this “game-changer” technology in ophthalmology will be addressed.

## 2. 3D Bioprinting for Eye Tissue Engineering

The eye is a very complex organ formed by different structures within which are the orbit, the sclera, the conjunctiva, the cornea, the iris, the pupil, the lens, the vitreous humor, the retina and the optic nerve [26]. Generally, each of the diseases that occur in the eyes affect only one of the aforementioned structures [27]. In this sense, the approximations carried out to date using 3D bioprinting have as their main objective the production of structures that resemble the characteristics and properties of the affected tissue. Such 3D-structures would allow the replacement of the entire damaged tissue or a part of it, in order to restore the patient’s vision. So far, the three main target tissues have been the cornea, the retina and the conjunctiva.

### 2.1. Cornea

**Figure 3:**
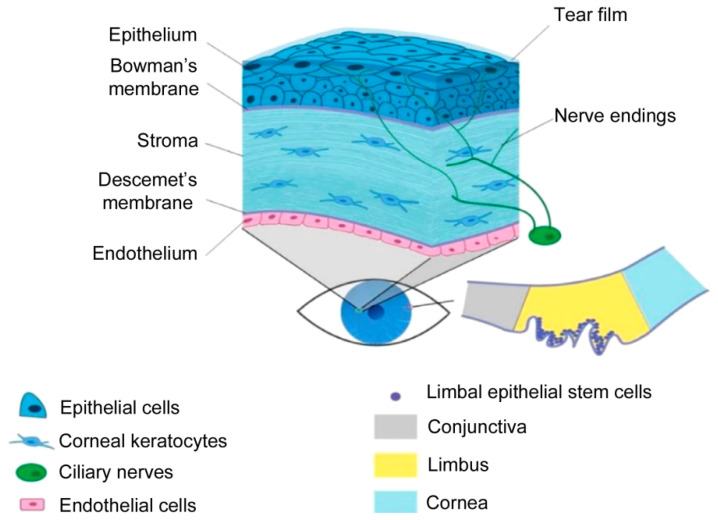
Anatomy of the cornea. Reproduced with permission from [20], Elsevier, 2019.

The cornea is an innervated and avascular tissue located in front of the pupil and iris [28]. Its main characteristic is its transparency, which allows the transmittance and refraction of the light entering the eye [20]. It acts as a mechanical and chemical barrier protecting the inner eye from external agents such as mechanical damage, microorganisms or ultraviolet radiation [29]. As a complex tissue, it is divided into five differentiated layers (Figure 3): epithelium, Bowman’s membrane, stroma, Descement’s membrane and endothelium [28,30].

The corneal epithelium is composed of a few layers of epithelial cells that form the outermost area of the cornea. The epithelial cells are constantly renewed from the basal layer, which is formed by limbal stem cells (LSCs) [31,32]. However, any damage in the basal area would lead to a dysfunction of the LSCs that would result in overgrowth of the conjunctiva and blood vessels, photophobia and pain [32]. The Bowman’s membrane is a thin acellular layer that separates the epithelium from the stroma [31]. The stroma is the widest part of the cornea. It is composed of laminin and collagen I fibrils that align perfectly to form a complex structure. This structural complexity is the key to its transparency and mechanical resistance [33,34]. In addition, it is composed of keratinocyte cells, which are responsible for maintaining the ECM [31]. These cells have little mitotic activity and present dendritic morphology. Nevertheless, in case of trauma, they are activated as a fibroblast that can differentiate into myofibroblasts [34,35]. Myofibroblasts express proteins that can alter the ECM causing opacity of the cornea, contraction and a corneal scar formation [31,35]. Descement’s membrane is an acellular layer that separates the stroma from the endothelium [31]. The endothelium is the deepest layer and is composed of endothelial cells that are responsible for maintaining the fluid balance of the cornea [31]. They have very little capacity of regeneration in vivo, therefore any damage in this area would cause irreversible blindness [36].

Taking into account the structural complexity of the cornea, degeneration of any of its parts could lead to serious diseases. In fact, bilateral blindness due to corneal damage has a high prevalence worldwide [29] and it is estimated that more than four million people suffer from a corneal disease [35]. With this demand, keratoplasty or corneal transplantation is the treatment of choice. However, the possibility of rejection, the poor survival of the explanted tissue, the absence of corneal banks, the high cost and scarce accessibility to transplantation necessitate the consideration alternative treatments [29,37].

Cell therapy [38] and the manufacturing of structures such as acellular membranes [39] have been proposed in order to supply high transplantation demand. However, it has been impossible to obtain a fully functional corneal substitute. In this context, 3D bioprinting has emerged as a promising technology since complex multilayer tissues, such as the cornea, can be easily reproduced. Furthermore, this technique can be advantageous over other techniques, since scaffolds with characteristics similar to those of the native cornea can be obtained. These characteristics are listed below.

Transparency. Transparency is its greatest characteristic. Previously proposed manufactured therapies, such as decellularized membranes, have had difficulties in achieving a transparency similar to that of the native cornea. As the key to transparency resides in a perfectly arranged structure, the use of 3D bioprinting that deposits layers exactly, as a pre-designed form, could be the solution.

Biomechanics. The biomechanical properties of the cornea affect corneal curvature, strength, and conformability [40]. Previously proposed treatments, such as cell therapy, have paid little attention to these parameters. The mechanical strength can be adjusted by combining different biomaterials. Conventional treatments, such as membranes, use a single material, thereby limiting the control of the biomechanical properties. On the contrary, 3D bioprinting is a technology that allows the use of a great diversity of materials with very diverse properties. In this way, the mechanical properties can be adjusted to the needs of the corneal tissue.

Curvature. Optical parameters of the cornea, such as light refraction, are due to its curvature [41]. Based on corneal geometrical information and using computer designed programs, a corneal prototype can be perfectly fabricated. This could be easily carried into the bioprinter achieving what would be hard to obtain with conventional techniques.

Multilayer structure. The cornea is a complex multilayer tissue. It is composed of different layers in which different materials, cells and internal structure are found. Conventional cell therapies have been shown ineffective due to the poor survival rate and functionality of the implanted cells [36]. Furthermore, single material membranes have not achieved the multilayer complexity of the cornea. Thus, 3D bioprinting overcomes this problem as it is based on the deposition of materials layer by layer. In addition, different cell types (epithelial, keratocytes and endothelial cells) can be embedded into biocompatible biomaterials increasing cell viability and functionality. As a result, a corneal native-mimicking structure with the five differentiated layers could be performed.

**Figure 4:**
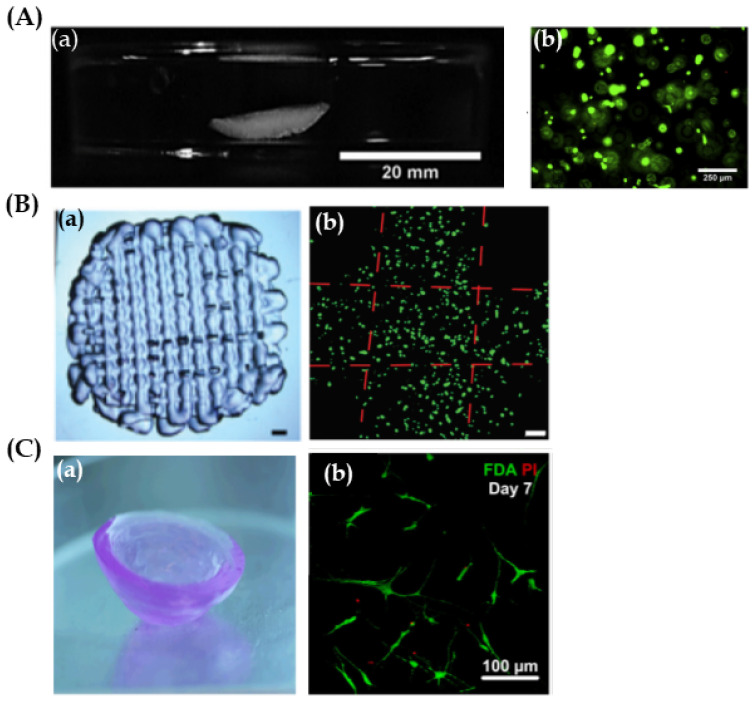
Corneal stroma structures and keratocytes. (**A**) Reproduced with permission form [42], Elsevier, 2018. (**a**) Images of extrusion bio-printed corneal stroma. Scale bar 20 mm. (**b**) Representative live/dead stain images using fluorescence microscopy after 3D bioprinting, showing live (green) and dead (red) cells. Scale bar 250 µm. (**B**) Reproduced with permission from [44], IOP Science, 2019. (**a**) Stereomicrographs of the methacrylate gelatin scaffold. Scale bar 1 mm. (**b**) Live/dead cell viability assay of 3D printed GelMA scaffolds on day 21, showing live (green) and dead (red) cells. Scale bar 100 μm. (**C**) Reproduced with permission from [37], Wiley, 2019. (**a**) Macrograph of ink-jet bio-printed 3D type I collagen/agarose scaffold. (**b**) Human corneal stroma keratocytes (CSK) stained with live/dead staining 7 days after bioprinting, showing live (green) and dead (red) cells. It is noticeable that the CSK is spreading and shows typical dendritic shapes in the scaffold. Scale bar 100 µm.

In this context, many studies have been carried out using 3D bioprinting technology for corneal tissue fabrication. Overall, these research studies have focused on the main part of the cornea, the stroma. Isaacson et al. [42] used extrusion based bioprinting for the development of a structure mimicking the stroma by embedding human keratocyte cells into an alginate/methacrylate type I collagen ink. They proposed the application of a rotational Scheimpflug camera in order to design patient-specific corneal model. Likewise, they developed a supportive structure to maintain the scaffold curvature during the bioprinting procedure. Consequently, they were able to reproduce the curved corneal geometry (Figure 4A-a) and cell viability was high for 7 days after bioprinting (Figure 4A-b). However, properties of great importance such as transparency and mechanical properties were not mentioned. These parameters were taken into a mixture of cells and ink placed onto slabs as a control. The study demonstrated that transparency and mechanical properties of bio-printed scaffolds were higher than those in the slabs, and data were similar to the native human cornea. Nevertheless, when analysing cellular studies, results were not as promising. Although cell viability was high in both systems, in both bio-printed scaffold and in slabs, cells were elongated and showed a dendritic morphology associated with keratocytes (Figure 4B-b). Therefore, cells’ metabolic activity and protein expression was low. It is believed that the high crosslinking density of 3D bio-printed scaffolds together with the absence of a curve geometry could negatively affect cell behaviour.

Kim et al. [43] studied this abnormal cell behaviour exhaustively after extrusion bioprinting. They wanted to analyze how the shear stress, applied when extruding through the printing nozzle, affects cell behaviour. To do so, they proposed to bio-print, by using different nozzle diameters, human keratocytes into a bio-ink based on decellularized corneal ECM in order to reproduce corneal stroma. Results demonstrated that not only shear stress affected cell behaviour, but also the deposition of collagen fibrils. In fact, by bioprinting with wide nozzle diameters, no aligned collagen fibrils were observed in scaffolds, which decreased transparency. Furthermore, cell dendritic morphology and keratocyte specific gene expression were not found. In contrast, after bioprinting using narrower nozzles, the shear stress increased and, as a result, highly structured collagen fibrils were shown. Nevertheless, cells were damaged and showed fibroblastic behaviour. The authors concluded that with the application of proper force, they could achieve scaffolds with both characteristics: structured collagen fibrils and cells with keratocyte behaviour. Thus, they bio-printed scaffolds with the proper extrusion nozzle (25G) and, after demonstrating that they met the adequate characteristics, scaffolds were implanted into rabbits. In vivo studies showed that implanted scaffolds were optically more transparent than the control (the not printed implant). In addition, keratocytes’ cellular behaviour was activated, which enhanced collagen production simulating a lattice pattern similar to the structure of native human cornea stroma [43].

In order to avoid keratocyte stress through the nozzle when extrusion bioprinting is used, Duarte et al. [37] proposed another approach; the use of the inkjet bioprinting technique. This study focused on bioprinting a stroma-mimicking structure by using type I collagen/agarose and human keratocytes bio-ink. After bioprinting, biomechanical properties, transparency, and cell viability and behaviour were analyzed. Results showed good printability using inkjet bioprinting, achieving curved and transparent scaffolds (Figure 4C-a). Moreover, cell viability was high. Rounded morphology was observed at day 1 after bioprinting. Nevertheless, cells became dendritic and showed keratocyte phenotype after 7 days (Figure 4C-b). In contrast, mechanical strength was lower than that of the human native cornea, so, improving mechanical properties would be their next main objective.

**Figure 5:**
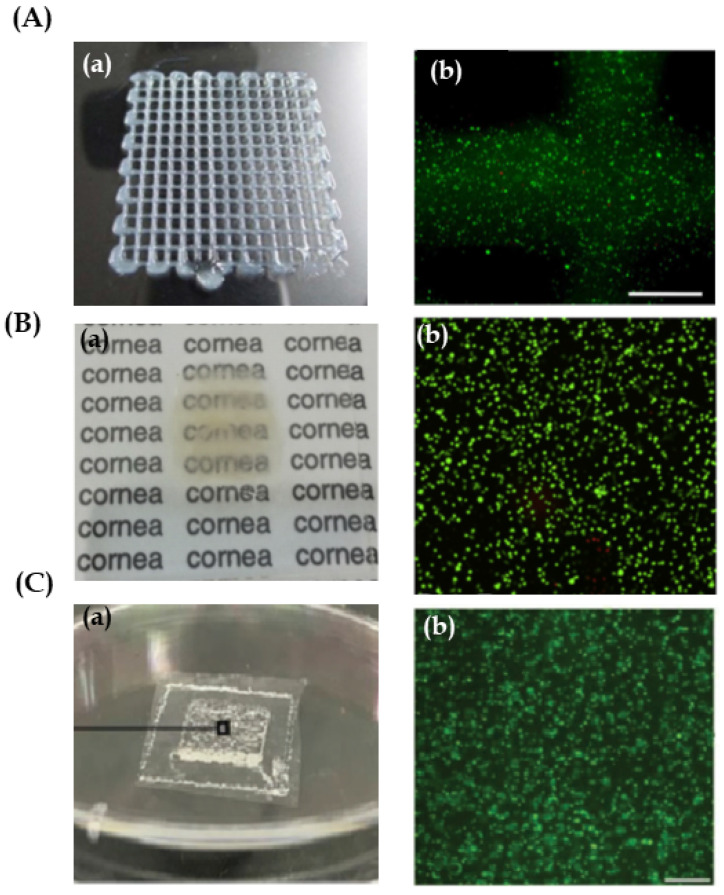
(**A**) Reproduced with permission from [45], Nature, 2016. Cornea epithelial structures: (**a**) Top view of a 3D human corneal epithelial cells/gelatin/alginate construct. (**b**) Epithelial cell viability after extrusion bioprinting by live/dead staining, showing live (green) and dead (red) cells. Scale bar 500 μm. (**B**) Reproduced with permission from [32], Elsevier, 2018. Cornea stroma and epithelium structure. (**a**) 3D bio-printed cornea from human embryonic stem cells and human adipose tissue derived stem cells fabricated onto supportive membrane using laser-assisted bioprinting. This shows moderate transparency. (**b**) Cell viability of human embryonic stem cells seven days after printing shown with live-dead-staining. Live cells are visualized with green and dead cells with red. Scale bar 1 mm. (**C**) Reproduced with permission from [46], Wiley, 2019. Cornea endothelium structures. (**a**) Image of the bio-printed genetically modified human corneal endothelial cells (HCECs)/gelatin scaffold on an amniotic membrane. (**b**) The seeded live HCECs were densely and evenly distributed just after bioprinting. Scale bar: 500 μm.

When the objective is to bio-print the corneal epithelium, fewer studies have been published so far. Wu et al. [45] proposed to bio-print human corneal epithelial cells embedded into alginate/gelatin ink, in which different concentrations of collagen I were added. Extrusion bioprinting was the technique that they selected in order to bio-print square grid-like scaffolds (Figure 5A-a). Results showed that the collagen concentration of 0.82 mg/mL was optimal for achieving scaffolds with high transparency and good resolution. In addition, cell viability was high after bioprinting, but cells showed rounded morphology (Figure 5A-b). The authors argued that cells embedded into alginate bio-inks were not able to degrade it and, therefore, they could not proliferate, elongate and differentiate. Thus, they proposed to add sodium citrate as a degradation system, so achieving controllable degradable scaffolds in which cell proliferation rate and epithelial specific protein expression were increased.

Another study was performed by Zhang et al. [30]. They focused on simulating a corneal structure by combining two manufacturing techniques, digital light processing (DLP) and extrusion bioprinting. They applied the first technique to create an acellular supportive corneal structure using methacrylate gelatin on which different concentrations of sodium alginate/gelatin ink mixed with human epithelial cells were deposited with the extrusion bioprinter. Parameters such as geometry, thickness, mechanical properties and transparency were analyzed. Besides, cell viability was assayed. Results showed that the DLP technique significantly improved the manufacturing accuracy. The geometry, curvature and thickness of the obtained scaffold were more similar to those of the native human cornea than those obtained in the other studies carried out so far. In contrast, extrusion bio-printed layers showed high diversity among alginate/gelatin bio-ink mixtures developed in this study, in terms of printability and mechanical properties, even though, the overall transparency was good. Cell viability was high but, as the authors focused mainly on improving the manufacturing precision and geometrical control, more in vitro studies should be carried out prior to taking these scaffolds into in vivo studies.

The previous studies made an approach towards manufacturing different parts of the cornea with different materials and techniques. However, to date, the only study in which the bioprinting of two different cellular parts of the cornea has been tried is that by Sorkio et al. [32]. They proposed the development of a corneal epithelium and a stroma using laser-assisted bioprinting (LaBP), and the use of stem cells, which show high differentiation capacity into epithelial stem cells and keratocytes. The authors argued that the LaBP technique may protect cells from the damage caused by nozzle stress. In addition, more viscous bio-inks can be used with this technique. In this study, two different bio-inks were developed, one based on human recombinant laminin, hyaluronic acid and human embryonic stem cells for corneal epithelium tissue, and another one composed of human collagen type I, blood plasma, thrombin and human adipose tissue derived stem cells for corneal stroma tissue. First, the scaffolds were bio-printed separately. Results showed that the bio-ink for bioprinting epithelium was printable using LaBP and that the cells embedded into the scaffolds showed high viability (Figure 5B-b). Moreover, epithelial cell morphology and expression of corneal epithelial markers were observed after 12 days. On the other hand, although the stroma-mimicking scaffold could be fabricated through LaBP without any difficulty, scaffolds lost their shape after a few days of culture, which indicated lost mechanical strength and the necessity of a supportive structure. Cells in the stromal scaffold showed high viability and expression of proliferative markers. In addition, cell organization resembled the native human corneal stroma. On the other hand, stroma scaffolds were implanted into explanted porcine eyes, which were maintained in culture, and a proper interaction and attachment to the host tissue was observed. Finally, a scaffold containing both layers, stroma and epithelium, was bio-printed in order to simulate native human cornea. The resulting structure was opaque due to the supportive membrane that was needed to avoid the stroma layer’s shape loss (Figure 5B-a). This opacity made its application difficult as cornea substitute as it did not meet with the main corneal characteristic of being functional. Thus, other supportive structures for the bio-printed corneal scaffold were needed in order to improve both transparency and stability.

Diseases affecting the corneal endothelium are the main cause of corneal transplantation [36]. Nevertheless, to date, only the study proposed by Kim et al. [46] has been focused on bioprinting. An interesting gene therapy approach was performed in which human corneal endothelial cells were genetically modified to express ribonuclease 5 (R5). R5 increases angiogenesis and facilitates endothelial cells’ mitotic capacity. Therefore, cell survival rate and wound healing ability are promoted. Cells were embedded into a gelatin ink and were bio-printed by extrusion onto a bovine decellularized amniotic membrane (AM) simulating the Descement’s membrane. After bioprinting, transparency, cell morphology and functional phenotype were assayed. Results showed that the scaffolds maintained their transparency for 10 days after bioprinting. Moreover, endothelial cells showed their usual shape and the R5 expression was high (Figure 5C). Then, scaffolds were implanted into rabbit’s cornea. Cell injection and an acellular membrane were used as controls. After surgery, there was an improvement in transparency in rabbits where a bio-printed scaffold had been implanted. Furthermore, four weeks later, levels of transparency near to normal cornea were achieved. In contrast, inflammation and persistent corneal oedema were observed in rabbits treated with cells and the acellular membrane. In addition, the endothelial corneal cells of the scaffold maintained their activity and shape, and rabbit native cell attachment to the scaffold was observed. Results were promising since they presented 3D bioprinting as a good alternative to the conventional treatments of corneal diseases.

As we have seen, to date advances have been made in the field of 3D bioprinting to create corneal tissue, and in most of them, extrusion bioprinting is the most commonly used technique (Table 1). However, it has been demonstrated that alternative techniques, such as inkjet or laser assisted bioprinting, could also be advantageous since they can in some cases be more cell friendly. The studies are recent and are focused on the most extensive layer of the cornea, the stroma. Even so, the development of corneal epithelial and endothelial tissues also seems quite promising. Until now, due to the complex characteristics of the cornea, it has not been possible to obtain a complete multilayer corneal tissue through 3D bioprinting. Nevertheless, this technique is in its beginnings and studies have shown interesting advances over common therapies. Therefore, more research should be carried out in order to achieve a functional corneal tissue. 

### 2.2. Retina

**Figure 6:**
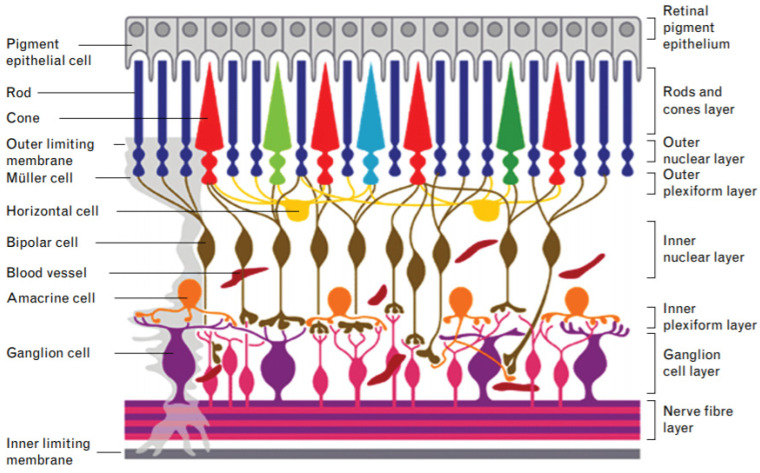
Diagram of the structure of the retina. The different cell types are located in different layers. The light reaches the nerve fiber layer. Ganglion cells transmit signals to bipolar and horizontal cells. Finally, they reach the rods and cones that transform it into electrical signals. The retinal pigment epithelium, formed by the epithelial pigment cells, served as physical, nutritional and signal support to the rest of the retina. Reproduced with permission from [53], Lippincott Williams and Wilkins Ltd., 2016.

The retina is a multilayered vascularized complex tissue situated at the back of the eye, opposite to the pupil [47] (Figure 6). Its main function is to convert the light signals that reach the eye into electrical signals that are conducted to the brain [48]. This function is possible thanks to the photoreceptor cells that make up this tissue. The retina is formed by more than 130 million cells of at least 60 different types [24,49]. They are generated from the fetal retinal progenitor cells [48]. Some of these cells are rod and cone photoreceptor cells, bipolar cells, horizontal cells, retinal ganglion cells and the glial cells, among others [50]. All these cell types work together to convert the light signals into electrical signals. Under the retina there is a monolayer known as the retinal pigment epithelium (RPE) formed by pigment epithelial cells. This specialized monolayer is a physical support for the retina. In addition, it provides nutrients and growth factors that create a suitable biological microenvironment for the cells of the retina [51,52].

The degeneration of the cells of the retina can lead to the appearance of different eye diseases. Some of these diseases are associated with a single cell type while others are associated with larger areas of the retina that involve different cell types. Some examples of the first type of disease are glaucoma [54], associated with retinal ganglion cells, and retinitis pigmentosa [55], associated with photoreceptor cells. An example of the latter is age-related macular degeneration (AMD) that arises because of mild and chronic inflammation of the central area of the retina [56]. The degenerations produced in the cells of the retina can lead to a progressive loss of vision until a total and irreversible loss is produced [27].

It is considered that many retinal diseases could be reversed if new cells from the retina were transplanted into the damaged area [57]. That is why many of the proposed therapies advocate the implantation of cells in the retina [58,59]. The administration of photoreceptor cells, progenitor cells, retinal sheets and RPE cells, among others, has been studied, although with not very clear results [60]. In fact, there is a considerable cell loss and a lack of control over cell behavior once implanted [61,62,63]. Therefore, appearance of abnormal behaviors and structures have been observed. As a solution to the problems involved in the implantation of cells in the retina, different scaffolds have been developed [64,65,66]. These scaffolds allow the transplantation of cells in a more controlled way. Solvent casting, electrospinning and molecular templating, among other techniques, have been used to produce these scaffolds [57]. Until now, none of the traditional microfabrication techniques have allowed the obtaining of 3D scaffolds with good structural properties and with the ability to incorporate the number of required cells in the correct position and orientation [24].

In this context, 3D bioprinting has been postulated as an excellent alternative for the design and production of scaffolds with characteristics that meet the needs of a tissue such as the retina. Some of the advantages that this technique offers are mentioned below.

Cell culture. It is very difficult to seed retinal cells in vitro [67]. Many of them undergo apoptosis and those that do not undergo dedifferentiation or stop producing the signals that they produce naturally in the eye [68]. Cells used for retinal regeneration have to be properly integrated and differentiated in the case of progenitor cells, or have to remain differentiated in the case of mature retinal cells. 3D bio-printed scaffolds allow the solving of these problems since they improve the viability and the maintenance of the phenotype of the implanted cells. Moreover, these 3D scaffolds provide mechanical and physical support for the adhesion, proliferation and differentiation of the retinal cells.

Complexity of scaffolds. The retina is a tissue with great structural complexity. It has several layers of diverse thicknesses and with different properties. 3D bioprinting allows the obtaining of precise scaffolds with highly complex designs (unlike previously used techniques that did not allow such control) [57]. The number of layers, their thickness and their spatial arrangement can be easily controlled by making a proper design and adjusting the printing parameters. It is necessary to replicate this spatial arrangement and thickness of the tissue so that the functions of the retina are not altered.

Cell types. The retina is a tissue with a high cell diversity. In particular, it has more than 60 different cell types [24,49]. 3D bioprinting allows the incorporation of different cell types to the scaffold [69]. These cells can be incorporated into different layers, thus resembling the original tissue. In this way, a possible approach could be the incorporation of ganglion cells in a first layer, bipolar cells in a second, cones and rods in a third, and RPE cells in a fourth layer. This arrangement would be very difficult to achieve by other current manufacturing techniques.

Cell orientation. The cells of the retina need a very specific orientation to be able to carry out their function properly [70,71]. The correct orientation of the cells is one of the most difficult aspects to achieve when making a scaffold. Although the new approaches that will emerge over time might allow more precise control of cell deposition, 3D bioprinting already allows a relative control over cell orientation. This can be achieved by adjusting the printing parameters, such as the printing orientation and layer thickness.

Stiffness. The average stiffness of the retina is 10–20 kPa [72]. Although this parameter is not as limiting as the previous limitations, it is important when trying to create a tissue to replace the one that is damaged [73]. 3D bioprinting works with a myriad of materials and their mixtures. This allows the achieving of a stiffness as similar as possible to that of the natural retina.

Taking into account all these advantages, different research groups have carried out approaches for retinal regeneration using 3D bioprinting and printing technology. Lorber et al. [74] used piezoelectric inkjet bioprinting to print retinal ganglion cell (RGC) neurons and retinal glial cells. The viability and effect of cell bioprinting on outgrowth in culture were studied. An abundant settlement of cells was detected in the nozzle. This fact significantly reduced the number of cells incorporated into the scaffolds. Nevertheless, the viabilities obtained were adequate compared to the control (69% and 78% for glial cells and 69% and 74% for retinal cells, respectively). Bioprinting did not appear to have a negative effect on the survival/regeneration properties of the cells in culture. Likewise, when used as a substrate, the glia cells that had been printed using 3D piezoelectric inkjet bioprinting retained their growth promoting properties. Kador et al. [60] used another 3D bioprinting technique to obtain their scaffold: the thermal inkjet bio-printing. In this study, they proposed a very novel approach since they evaluated the possibility of bioprinting RGC cells on an electro-spun matrix of polylactic acid. Different parameters were modified during bioprinting, such as the ejection energies and the cell densities. The results were promising. As in the previous study, good cell viabilities were obtained indicating that thermal inkjet bioprinting can effectively be used for obtaining scaffolds for retina regeneration. Furthermore, the bio-printed RGCs maintained adequate electrophysiological properties. One of the main goals of this study was to achieve a proper orientation of the cells. The microscopy images showed how the design of the matrix and the 3D bioprinting allowed the achievement of a radial arrangement of the axons of the printed cells. In this way, the orientation of the RGC was significantly improved compared to the control. Specifically, 72% of the axons were aligned with the scaffold while, in the case of the dendrites, 49% were aligned. Only 11% of the cells of the control group were aligned.

**Figure 7:**
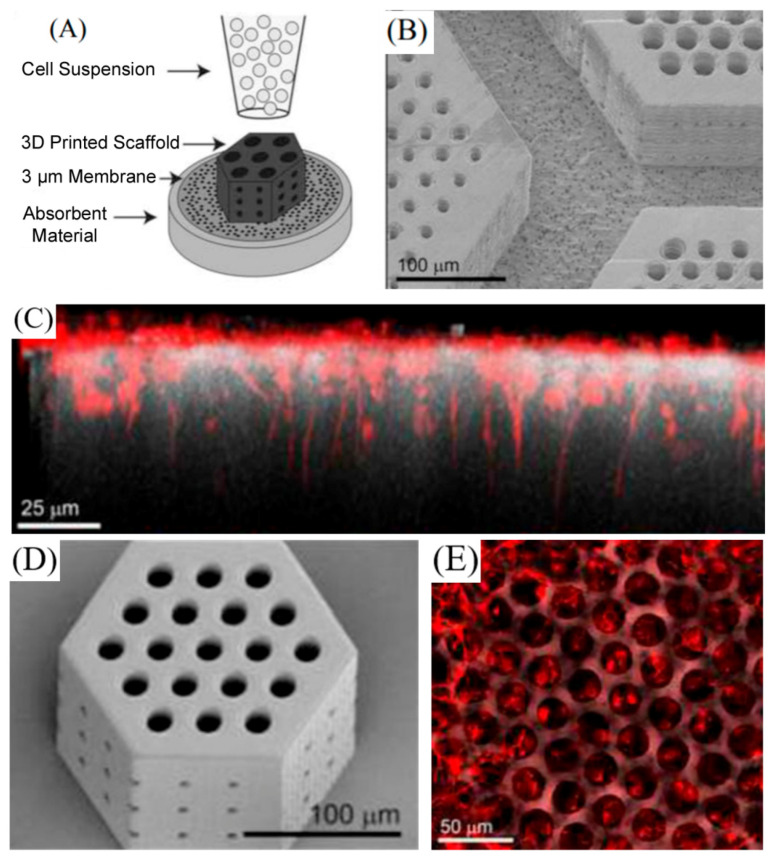
(**A**) Schematic of the retinal progenitor cell loading strategy. (**B**) Large photoreceptor cell porous membrane adhered to the membrane after processing. (**C**) Side view of retinal neurons (marked in red) settled in and aligned with 25 μm vertical pores. (**D**) Representative scanning electron microscopy (SEM) image of a small retinal progenitor cell scaffold used to determine design-to-structure fidelity. (**E**) Sequential top-down images of retinal neurons (marked in red) on the surface of photoreceptor scaffolds and nestled in 25 μm pores. Reproduced with permission from [57], Elsevier, 2017.

Another study in which great importance was given to the orientation of the cells was that carried out by Worthington et al. [57]. In this study, different scaffolds using two photon polymerization were obtained. The studied variables were the pore size, the hatching distance, the hatching type and the slicing distance. The time necessary for printing and the fidelity of the obtained scaffolds with respect to the original designs were analyzed and optimized. In addition, induced pluripotent stem cells (iPSCs) were differentiated into retinal progenitor cells and incorporated into the printed scaffolds (Figure 7). The obtained results made it possible to clarify that those scaffolds with larger diameter pores were better for use in retinal regeneration. In these scaffolds, retinal progenitor cells could be incorporated. Besides, cells formed neural structures aligned in parallel to the vertical pores of the scaffolds. In contrast, in the scaffolds with smaller diameter pores, cells remained on top of the surface and did not align in parallel to the pores.

**Figure 8:**
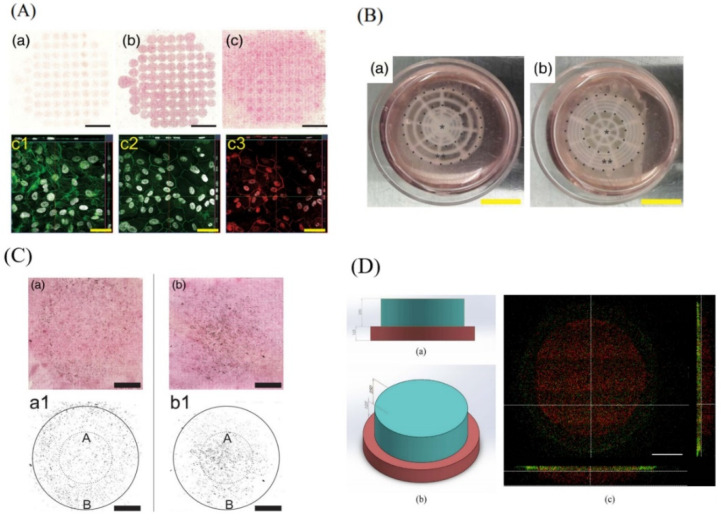
(**A**) Bio-printed ARPE-19 cells (**a**–**c**): hematoxylin and eosin staining at days 1, 7, and 14, respectively, scale bar: 5 mm. Confocal images of ARPE-19 cell layers at Day 14: F-actin cytoskeleton immunofluorescence staining (**c1**), Zonula occludens-1 (**c2**) in green, Claudin-1 (**c3**) in red, and nuclei in grey, scale bar: 50 μm. Reproduced with permission form [75], Wiley, 2018. (**B**) The bio-printed retinal equivalents with two distinctive Y79 cell-seeding density: high average cell density at the center (**a**) and high average cell density at the periphery (**b**); *: central area, **: periphery; scale bar: 10 mm. Reproduced with permission from [24], Whioce Publishing Pte. Ltd., 2017. (**C**) Hematoxylin and eosin staining of the bio-printed Y79 cells with two distinctive patterns on ARPE-19 cell monolayer at Day 14: high density at periphery (**a**) and high density at the central area (**b**). Data analysis (integrated intensity) of bio-printed Y79 proliferation after 14 days, high density at periphery (**a1**), and high density at the central area (**b1**); A = area of the inner circle; B = area of the annulus; diameters: small circle (10 mm) and large circle (20 mm); scale bar: 5 mm. Reproduced with permission from [75], Wiley, 2018. (**D**) Bilayer printing of fluorescent labelled hydrogels: (**a**) Top view and (**b**) side view of structural design from SolidWorks^®^ (**c**) Confocal fluorescent images showing bilayer construct. The printed structure recapitulated the structural design, indicating ability to construct multi-layered structure. Scale bar = 500 µm. Reproduced with permission from [76], Elsevier, 2018.

Other studies have focused their attention on reproducing the structure of the retina. They have proposed different approaches to obtain different layers seeded with different cell types. Two examples of this type of approach are the ones proposed by Shi et al. [24,75]. Using microvalve-based bioprinting, a structure equivalent to that of the retina was created. A first monolayer was printed, made up of alginate and pluronic-containing RPE cells (ARPE-19) on a preformed membrane (Figure 8A). This structure simulated the RPE monolayer. Over it, a second layer was bio-printed. This was also made up of the same materials and contained photoreceptor cells (Y79) (Figure 8B,C). Two types of pattern were created for the top layer, one with a higher density of cells in the center and another with a higher density at the perimeter. The bio-ink printed with Y79 cells preserved its structure during the culture process. Viability was not compromised and cell density increased over time. This proof of concept demonstrated that a structure with characteristics similar to those of the retina can be obtained, achieving good cell viability and cytocompatibility. Wang et al. [76] made a similar approach. In this case, the 3D bioprinting technique used was laser assisted 3D. They obtained a two-layer scaffold similar to the retina. The bio-ink used in both layers was the same HA-GM (hyaluronic acid with methacrylation by glycidyl-hydroxyl reaction) and polyethylene-glycol-Arg-Gly-Asp-Ser peptide (PEG-RGDS). The difference between the two layers lay in the thickness and in the incorporated cells. For the RPE layer, RPE cells and a thickness of 125 um were used, while for the upper layer fetal retinal progenitor cells (fRPCs) were used that were differentiated to retina photoreceptors (PR), along with a layer thickness of 250 um. The porosity of the scaffold was analyzed as a function of the degree of methacrylation of the hyaluronic acid (low, medium or high), the swelling ratio, the rigidity of the scaffold, the viability of the cells, the formation of the two layers in the scaffold and the differentiation of fRPCs cells into PR cells within the scaffold. Results were encouraging since the stiffness of the scaffold was similar to that of the native retina, the viability remained above 70%, the microscopy images showed that two well-differentiated layers had been obtained (Figure 8D), and the fRPCs did differentiate into PR. Therefore, it was concluded that tis co-cultivation system allowed the development of an environment similar to that of the native retina, which promoted the maturation of PRs.

To date, these studies have made it possible to determine: (i) the cell viability after printing; (ii) the structure of the scaffolds; (iii) the orientation of the cells within the scaffolds; and (iv) their arrangement in different layers (different levels or heights) (Table 2). Still, much more research is needed. Among the steps to be taken in the near future are the bioprinting of more layers, the use of more cell types and the study of the ability of the bio-printed cells to transmit signals within the scaffold itself. In addition, it is expected that in the future the scaffolds obtained using 3D bioprinting will be more complex and similar to the native retina. Finally, these bio-printed tissues will serve as autologous retinal cell grafts to treat those patients suffering from retinal degeneration. 

### 2.3. Conjunctiva

The conjunctiva is a mucosal tissue that covers the sclera and provides lubrication and protection to the eye by producing tears and mucus [77]. It consists of a goblet cell rich in highly vascularized stratified epithelium [78]. This tissue suffers from different injuries caused by ocular thermal or chemical abrasions, conjunctival lacerations, autoimmune diseases, inflammation, foreign bodies or surgery, etc. [79]. To treat the damaged conjunctiva the usually employed strategies are surgery and autologous grafts or allograft tissues such as AM and pericardium [80]. These approaches have some limitations: (i) unavailability of healthy conjunctiva (in the case of autologous grafts); (ii) immune responses; (iii) keratinization; (iv) goblet cell loss; (v) microbial infections; (vi) low level of stratifications, and (vii) opacification of the site, etc. [78]. These limitations make necessary the development of new strategies among which 3D printing has been postulated as an excellent alternative. 3D printing can act as an improvement for the development of structures similar to the conjunctiva as it allows the achievement of the following characteristics.

Thickness. It is crucial to develop a 3D structure with an adequate thickness; that is, as close as possible to the original (average thickness of 33 μm). By determining the number of layers and their thickness, the size of the 3D printed structure can be controlled very precisely.

Cell density. 3D bioprinting allows control of the density of goblet cells included in the 3D developed membrane.

Transparency, elasticity and slight rigidity. The large number of materials that can be used in 3D bioprinting allows the controlling and adjusting of the color and transparency of the construct. These materials also make possible the obtaining of elastic properties similar to that of the healthy tissue. In addition, the rigidity of the scaffold can also be adjusted. One of the problems related with the grafts used so far is the difficulty of handling in operations because of their fineness. 3D bioprinting makes it possible to achieve scaffolds with sufficient rigidity to facilitate manipulation, without this, in turn, having a negative biological effect.

Biological activity. The use of materials of biological origin allows the adjustment of the re-epithelization capacity, decreasing scar formation and fornix foreshortening, adjusting biodegradability and achieving good biocompatibility.

So far, the only approach that has used 3D printing to obtain a structure that allows regeneration of the damaged conjunctiva is the one carried out by Dehghani et al. [81]. They developed a membrane by extrusion 3D printing using gelatin, elastin and hyaluronic acid as materials. They carried out multiple rheological and texturometry tests to guarantee that the ink and the membrane obtained had the necessary properties to be implanted in the conjunctiva. Biological properties were also analyzed in terms of cytocompatibility, adhesiveness and cell proliferation in vitro, and epithelialization, inflammation, scar tissue formation and presence of granulation tissue in vivo. All these results were compared with an AM (frequently used in injuries of conjunctiva). The results obtained were promising. The ink could be properly printed and the obtained membrane had adequate color and transparency, and its handling was simpler than that of the AM. Furthermore, in vitro good cytocompatibility, adhesion and cell proliferation were obtained. In vivo, the results were also positive. The epithelization time was similar in the printed membrane and in the AM. However, the data regarding inflammation, cell density, degradation and granulomatous reaction were better in the 3D printed membrane group.

These results show that this technique is suitable for the development of membranes for the regeneration of damaged conjunctiva. In this sense, it is reasonable to think that in the near future more research into the application of 3D printing to conjunctiva regeneration will be carried out.

## 3. 3D Printing for Ocular Drug Delivery

The main objective in the field of pharmacology is to achieve the maximum therapeutic effect with the minimum toxicity [82]. In this regard, the development of personalized patient specific drugs or doses is booming. 3D printing has recently been postulated as the appropriate technology to accomplish this goal due to its ease of use, fast speed, and accessibility [83]. In this way, the possibility of developing pharmaceutical forms containing various drugs, adjusting the doses to each patient and modifying drugs’ pharmacokinetic profiles has become a reality in preclinical studies thanks to 3D printing technology [83]. This personalized medicine would bring a huge advantage to patients suffering from chronic eye pathologies, since current treatments require the constant application of eye drops and, in the worst cases, repetitive intravitreal injections that can cause devastating intraocular inflammation [84].

In this context, although there have been several studies in which 3D printing devices have been developed as drug delivery systems, only the one proposed by Won et al. [84] focuses on the eye. In this study, a flexible coaxial printing was used in order to develop a system that contained two drugs for the treatment of retinal vascular disease (RVD), which is based on an abnormal vascularization of the retina. The drug delivery system consisted of an external shell composed of polycaprolactone and bevacizumab (PCL-BEV), a drug that prevents excessive angiogenesis. The interior part was composed of sodium alginate and dexamethasone (ALG-DEX), an anti-inflammatory drug. In vitro assays showed a continuous release of BEV and DEX. Moreover, good biocompatibility and a reduction in the growth rate of human umbilical vein endothelial cells was observed. Then, the printed drug delivery system was intravitreally injected into two animal models: rabbits, in order to study the release kinetics, and choroid neovascularization (CVC) rat models, to determine its therapeutic effects. Intravitreal drug injections were used as control. Results corroborated that the printed drug delivery system prolonged the release of BEV and DEX compared to control. Interestingly, higher angiogenic inhibition over time was observed in CVC rats compared to controls and a reduction of inflammation was achieved.

As reflected in this study, the implementation of 3D printing technology for the development of specific drug delivery systems in ophthalmology can be valuable. In addition, the manufacture of artificial tissues similar to native tissues with this technology can be useful when it comes to screening new drugs for eye diseases. However, before this technology can be extensively applied in clinics, multiple regulatory questions should be addressed.

## 4. Ethical Issues and Commercialization Regulatory Aspects

Although the previously mentioned technical requirements can be fulfilled by increased research knowledge, there are still some relevant concerns related to both ethical and regulatory aspects that jeopardize the road to clinic of this “game changer” technology [1,2].

From an ethical point of view, a clear benefit of this technology includes the use of 3D bio-printed ocular organs or tissues for academic or research applications, as intermediate drug testing models between in vitro conditions and in vivo probing of concepts. Such models could work as a promising alternative to minimize the use of animals in the laboratory. In addition, patients’ own cells reprogrammed into induced pluripotent stem cells (iPSCs) and bio-printed into 3D ocular structures could represent a more efficient and reliable model for drug testing than the use of experimental animals. However, there are also risks and ethical issues that should be considered, especially when 3D bioprinting technology is aimed at tissue engineering purposes. In this case, the composition of biological inks raises some concerns, not only associated with the security of the grafts implanted, but also related to the biological origin of such cells [3]. In the clearest scenario, where patient’s autologous cells are included in the bio-ink composition, a random migration of cells from implanted ocular grafts could arise in different parts of the body, leading to potential undesired effects [5,8]. It is also likely that the biological behavior of such cells can be altered due to the mechanical stress that cells suffer by the transient forces applied during the bioprinting process. Therefore, although 3D bioprinting technology holds huge potential in tissue engineering clinical practice, the benefit-risk balance should be considered, in the same way as in other advanced therapies such as cell and gene therapy. In case non-autologous cells are implanted, apart from previously mentioned concerns, the possibility of inflammatory response against implanted ocular graft also needs to be analyzed. In this scenario, it should not be forgotten that the donor of cells for the implementation of 3D bio-printed grafts needs to sign an informed consent to allow the use of such cells. The situation is more delicate if non-autologous stem cells at the embryonic status are manipulated and bio-printed to become part of a grafted ocular tissue or organ, due to the ethical dilemma that arises with the use of embryonic cells. Another more challenging possibility is the use of cells derived from animal models to print ocular tissues implanted in humans. In this case, in addition to the previously mentioned biological concerns, the risk of developing zoonosis diseases should also be considered [20].

However, not only the origin and status of bio-printed cells rise ethical issues. We should also consider if any biological “item” can be printed [21]. For instance, and leaving apart any technological consideration, strictly from an ethical point of view, when implanting bio-printed tissues, a retina can be more problematic than a cornea, due to the neuronal structure of the retina and its direct connection to specific areas of the brain. This issue also generates the discussion as to whether specific areas of the brain, or even the brain or the eye as a whole, could be bio-printed and implanted into human beings.

Another ethical concern for the application of 3D bioprinting technology into regular clinical practice arises from the design and implementation of clinical trials for personalized medicines. Traditionally, clinical trials are designed and classified into different phases to evaluate the safety of the treatment in the early stages, and later the efficacy in a large population, before the introduction of a drug into the market. However, this approach is not feasible in the case of patient-tailored medical products for ophthalmic purposes, due to interindividual variability among human beings, which hampers any extrapolation of the results obtained in a specific patient, although experience accumulated in clinical cases could serve to gain progressive knowledge in order to apply the technology in medical practice [22]. Furthermore, the irreversible nature of grafts implanted by bioprinting technology impedes the patient’s withdrawal from the trial after implantation, in the case of complications. In this sense, the development of 4D bio-printable organs, that can be biodegraded under physiological conditions after having performed the desired effect, merits special attention [24,25]. However, it should be also borne in mind that enrolling in this kind of clinical trial involves the acceptance of high risks associated with the implementation of the technology. Therefore, only advanced stages of diseases that have been unsuccessfully treated with conventional approaches should be amenable for this kind of treatment.

When dealing with health and financial resources, it is logical to expect ethical conflicts. In this sense, the technological possibility to bio-print and implant specific organs or tissues as an alternative solution to face advanced stages of diseases could stratify society. In this sense, even though the manufacturing of bio-inks and 3D bio-printing are not expensive items, the multidisciplinary nature of the global process, as well as the surveillance of this approach, makes it a high-cost procedure [85]. Hence, its implementation would be not affordable for all strata of society, only being obtainable for a particular subgroup of the total population with access to financial resources. Therefore, only those who can afford to pay for the bio-impression of their own organs would presumably enjoy a longer and better quality of life, minimizing for instance the use of immune-suppressant drugs indicated to avoid rejections of conventionally transplanted organs. In addition, relevant organs or tissues specifically designed with better biological properties could be implanted not only to deal with advanced stages of diseases, but also to enhance their physiological performance, which at the end could result in the elaboration of “super” bio-items for eugenic goals [86], such as “super” eyes or retinas.

Considering all the risks and benefits that 3D bioprinting technology can offer in the coming years, not only to the research and medical community but also to patients affected by ocular diseases, legal and regulatory aspects related to the implementation of the technology need also to be also deeply analyzed to avoid illicit and fraudulent use if the technology finally ends up in the “wrong hands”. However, actually, the scale-up of 3D bioprinting technology and its clinical application for medical purposes does not fit into any of the current regulatory categories, despite that, from a global point of view, it could be considered as a specific tissue engineering approach [87]. In addition, the patient-tailored application of the technology hampers the compliance with global regulatory requirements for commercialization purposes that at moment are limited to recommendations, notifications and reports provided by European and American agencies, the EMA and FDA, respectively. Although nowadays both agencies aim to legislate the application of this innovate technology into clinical practice, they lack a specific regulatory framework [88]. From a more world-wide point of view, only a few regulatory agencies in countries such as Japan and South Korea have developed broad regulatory measures that can be applied to 3D bioprinting technology. This initiative could work as a starting point and reference for other countries. In any case, such regulatory measures are mainly focused on the application of these technologies for academic and research purposes or for the development of acellular devices in ophthalmology such us spectacles, lenses or smartphone-based fundus cameras, etc. [88]. Therefore, in order to implement this technology regularly in medical practice, it is necessary to start working on the design of a multidisciplinary and world-wide panel to provide a global regulatory framework. It also should be borne in mind that, in order to find a solution and resolve legal problems associated with the technology, all related tenets need to be addressed. In this sense, due to the complexity of the technology, a “whole” legal approach would be preferred, rather than a “piecemeal” approach.

## 5. Current Challenges and Future Perspectives of Ocular 3D Bioprinting

Recent advances in ocular 3D bioprinting have brought new opportunities for eye tissue engineering with potential biomedical applications, and nowadays it seems unquestionable that this field will continue to grow and evolve in future years. However, there are still relevant challenges to overcome before ocular and, in general, 3D bioprinting becomes a real clinical option.

First, the materials for bio-ink preparation should be biologically functional while maintaining robust and controllable post-printing mechanical properties and should enable adequate physiological, bio-chemical and mechanical interactions with the cellular component [89]. One strategy to find a desirable balance between biological activity and mechanical characteristics is the use of hybrid constructs that contain, on the one hand, synthetic materials that provide structural integrity and, on the other hand, natural materials that provide a cell-friendly environment for cellular growth. Other approaches are based on the chemical modification of the scaffold or on the use of synthetic peptides such as Arg-Gly-Asp (RGD) in order to prompt the crosslinking of the material and control its mechanical properties or its degradation time. For instance, a similar strategy was used for retinal bioprinting using a bio-ink based on HA with methacrylation by glycidyl-hydroxyl reaction and PEG-RGDS, with encouraging results, since the mechanical properties achieved allowed the bioprinting of two retinal layers with suitable rigidity and high cell viability [76]. In addition, cell differentiation occurred within the scaffold, which indicated that the hydrogel was biologically active and enabled biochemical interactions with the cell component. In this regard, appropriate cell orientation within the scaffold has also been achieved in retinal bioprinting using retinal ganglion cells that maintained their functional electrophysiological properties after being printed, demonstrating that the scaffold provided suitable physiological, biochemical and mechanical interactions for that purpose [60].

Cell sourcing constitutes another important challenge for 3D bioprinting of tissues and organs. In fact, a high number of regeneration-competent cells are needed and, due to tissue heterogeneity, different types of cells are also required. In fact, most organs are more complex than current 3D bioprinters can reproduce and achieving their intricate composition and functionality is still far from clinical translation [4,90]. In eye 3D bioprinting, most ocular structures bio-printed so far are limited to one or two cell types and, in the case of cornea and retina, only one or two layers have been bio-printed in the same construct, which is far from the authentic complex configuration of these ocular structures. In any case, regarding the challenges related to limited cell availability in forming the scaffolds, some of the solutions carried out to date are based on the use of stem cells or progenitor cells such as mesenchymal cells or induced pluripotent stem cells (iPSC), which, ideally, should be autologous or non-immunogenic [89]. In this sense, human fetal retinal progenitor cells have been used for retinal bioprinting and successful differentiation into photoreceptor cells was achieved [76]. In addition, human iPSC have also been used for retinal bioprinting and differentiated into retinal progenitor cells [57]. Considering all this, important advances have been made in order to overcome specific challenges related to cell sourcing, but more fundamental research is still needed in order to be able to bio-print a whole functional ocular structure such as the cornea or the retina with all the required different cell types and layers and the necessary biomechanical cues. These advances would contribute in the future to mimicking the complexity of the desired organ structures in a more precise manner.

In addition to the difficulties in obtaining a highly complex and similar to native bio-printed construct, another key aspect to consider before clinical translation could occur would be the vascularization and innervation of such tissue-engineered constructs after transplantation. So far, most tissue constructs obtained by 3D bioprinting lack a functional vascularization network, which would hinder the oxygen and nutrient supply after implantation in vivo [89]. Different strategies have been postulated in order to solve this issue in different types of tissue, including the use of angiogenic growth factors, the embedding of microchannels that would enhance the diffusion of oxygen and nutrients, or the direct fabrication of vasculature [89]. However, the reproduction of the complex and complete vascular network necessary for clinical translation remains very challenging. Regarding the eye, bioprinting a full ocular structure has still not been accomplished, so that would be the first step before moving to vascularization and innervation of the tissue construct. However, this aspect would acquire special relevance in the case of the retina, where the light signals captured by the photoreceptors must be processed into electrical impulses and transmitted through the optic nerve to the brain. In this regard, the functional electrophysiological properties of bio-printed retinal ganglion cells achieved so far [60] hold promise for future functional retinal constructs with appropriate connections among cells of the different retinal layers, capable of completing the complex visual phototransduction process.

In summary, recent advances in the field of 3D bioprinting and, particularly, for ocular tissue-engineering show promise for future biomedical applications, although there are still many challenges that need to be overcome before clinical translation can occur. Mimicking the complexity and heterogeneity of organs and providing them with the diversity of functional and supporting cell types, as well as with the essential functional elements such as vasculature and innervation, represent the major difficulties that 3D bioprinting is currently facing. In addition, for future commercialization, further aspects such as standardization of protocols, regulation of the process, the cost-effectiveness for scaling and the logistics of 3D bio-printed products would need to be taken into consideration. The key benefits of 3D bioprinting include the possibility of more targeted and personalized medicine, automated tissue fabrication and the flexibility of incorporating a wide variety of cells and materials in a precise anatomical 3D geometry. Further research for in vitro optimization and in vivo implementation of bio-printed tissue constructs and the combined efforts of different multidisciplinary fields would enhance the progress of 3D bioprinting towards clinically relevant bio-printed organs.

## 6. Conclusions

In recent years, great advances have been made while using 3D bioprinting for eye tissue engineering. Different ocular tissues with various layers and various cell types have been replicated. Nevertheless, we are still far from achieving complete tissues similar to healthy ones. It is expected that, in the future, the different ocular components will be completely replicated using 3D bioprinting. This progress could allow the combination of the structures obtained and to integrate them into a single construct, as a complete ocular model [27], which would require the consideration of corresponding ethical and biological aspects. Furthermore, as previously mentioned, the generated models would allow planning of operations before performing them, understanding the interaction between cells and the progression of different diseases affecting the tissue, or analyzing the effects of diverse drugs.

## Figures and Tables

**Table 1:** Current studies of 3D bioprinting for cornea tissue engineering.

3D Bioprinting Technique	Materials of the Bio-Inks and Inks	Cells	Scaffold Function/Study Objective	In Vivo	Most Relevant Results	Ref.
Extrusion 3D bioprinting	Sodium alginate and methacrylated type I collagen	Human corneal keratocytes	Tissue replication. Corneal stroma structure	No	Reproduce corneal curvature Good printability High cell viability after 7 days of bioprinting	[42]
Extrusion 3D bioprinting	Methacrylated gelatin (GelMA)	Human corneal keratocytes	Tissue replication. Corneal stroma structure	No	Excellent transparency Adequate mechanical strength High cell viability but rounded morphology and low metabolic activity	[44]
Extrusion 3D bioprinting	Decellularized corneal extracellular matrix based bio-ink	Human corneal keratocytes differentiated from human turbinate derived mesenchymal stem cells	Tissue replication. Corneal stroma structure	New Zealand white rabbits	Establishment of the best nozzle diameter in order to bio-print aligned collagen fibrils similar to cornea Establishment of the best nozzle diameter in order to maintain keratocyte morphology and phenotypic characteristics Transplanted scaffold showed good transparency in rabbit eyes Keratocytes’ cellular behaviour was activated after transplantation	[43]
Drop-on-demand inkjet bioprinting	Type I collagen and agarose	Human corneal keratocytes	Tissue replication. Corneal stroma structure	No	Good transparency and optical density but low mechanical properties Good cell viability. Cells became dendritic and achieved typical keratocyte shape. Cells maintained their phenotype after bioprinting.	[37]
Extrusion 3D bioprinting	Sodium alginate, gelatin and type I collagen	Human corneal epithelial cells	Tissue replication. Corneal epithelium structure	No	Good printability and high transparency High cell viability after bioprinting but round morpholog Fabrication of degradation-controllable systems using sodium citrate Improvement of cell proliferation, growth and epithelial specific marker protein expression with the degradation system	[45]
Combination of digital light processing (DLP) and extrusion 3D bioprinting	Methacrylated gelatin (GelMA) for DLP Sodium alginate and gelatin for extrusion 3D-bioprinting	Human corneal epithelial cells	Tissue replication. Development of supportive structure with DLP technique in order to bio-print corneal epithelium structure on it	No	Good development of cornea structure with digital light processing (DLP) in terms of geometry, thickness and curvature. Overall, good transparency of epithelium scaffolds but high diversity in mechanical properties High cell viability and distribution	[30]
Laser-assisted 3D bioprinting	2 Types: Human recombinant laminin and Hyaluronic acid sodium Human collagen type I and Human blood plasma + Thrombin	Human embryonic stem cells (hESC) Human adipose derived stem cells (hASC)	Tissue replication. Cornea epithelium structure Corneal stroma structure	No Explanted porcine corneas	Good printability with laser-assisted bioprinting High hESC viability and epithelial specific marker protein expression High proliferative protein expression in hASC Strong adhesion, cell migration and good attachment to the host tissue in explanted porcine corneas Opacity when both layers were combined	[32]
Extrusion 3D bioprinting	Gelatin based bio-ink	Human corneal endothelial cells genetically modified to express ribonuclease (R5)	Tissue replication. Corneal endothelium structure.	New Zealand white rabbits. Descemet’s membrane-denuded corneal disorder model.	High transparency High cell viability, usual endothelial shape and high R5 expression. Improvement of rabbit corneal transparency in vivo. High functional phenotype expression and native cell attachment in vivo.	[46]

**Table 2:** Current studies of 3D bioprinting for retina tissue engineering.

3D Bioprinting Technique	Materials of the Bio-Inks and Inks	Cells	Scaffold Function/Study Objective	In Vivo	Most Relevant Results	Ref.
Laser assisted 3D bioprinting	HA-GM (hyaluronic acid with methacrylation by glycidyl-hydroxyl reaction) and PEG-RGDS (Arg-Gly-Asp-Ser peptide)	Retinal pigment epithelial cells (RPE) Human fetal retinal progenitor cells (fRPCs)	Tissue equivalent replication. Retina made up of two layers	No	Development of a structure of two layers: one assembling the retina (using fetal retinal progenitor cells (fRPCs)) and the other assembling the pigment epithelium (using RPE) Good cell viability Differentiation of fRPCs to PRs (photoreceptor cells) within the scaffold	[76]
Piezoelectric inkjet bioprinting	DMEM (Dubelcco’s Modified Eagle’s Medium) (not structural function)	Retinal ganglion cell (RGCs) neurons Retinal glial cells.	Study the effect of piezoelectric inkjet bioprinting in the viability of the printed cells.	No	Piezoelectric inkjet allows printing of retinal cells with similar survival/regeneration properties to controls Printed glial cells retain their growth promoting capability when used as substrate Cell sedimentation occurred in the nozzle area	[74]
Microvalve-based inkjet bioprinting	DMEM:F12 (not structural function) Alginate and Pluronic	Human retinal pigmented epithelial cell line (ARPE-19) Human retinoblastoma cell line (Y79)	Tissue replication. Retina made up of two layers.	No	Development of a structure formed by a monolayer with ARPE-19 cells (representing the Brunch’s membrane and the RPE monolayer) and a second with a human retinoblastoma cell line (Y79) The obtained structure is stable Viability is not compromised and cell density increases with time	[24]
Two-photon lithography	Indium tin oxide (ITO)-coated glass	Human induced pluripotent stem cell (iPSC)	Development of scaffolds to deliver correctly oriented retinal progenitor cells	No	Establishment of the best parameters to print scaffolds using two-photon lithography with adequate and reproducible characteristics Differentiation of iPSCs to retinal progenitor cells and incorporation of these last into the scaffold The retinal progenitor cells formed neural structures parallel to the vertical pores of the scaffolds	[57]
Thermal inkjet 3D bioprinting combined with electrospinning	Alginate and culture Medium for 3D bioprinting Polylactic acid (PLA) dissolved in 1,1,1,3,3,3 hexafluoro-isopropanol (HFIP) and matrigel for electrospinning	Retinal ganglion Cells (rgcS)	Development of scaffolds to deliver correctly oriented retinal progenitor cells	No	Determination of printing parameters, materials and cell density in order to print a pattern with an organization similar to that of the human retina Good cell viability, adequate orientation of the cells in the pattern and correct guidance of the axons within the scaffold The cells maintained their functional electrophysiological properties after being printed	[60]
